# Rats Prone to Obesity Under a High-Carbohydrate Diet have Increased Post-Meal CCK mRNA Expression and Characteristics of Rats Fed a High-Glycemic Index Diet

**DOI:** 10.3389/fnut.2015.00022

**Published:** 2015-07-09

**Authors:** Catherine Chaumontet, Dalila Azzout-Marniche, Anne Blais, Tristan Chalvon-Dermersay, Nachiket A. Nadkarni, Julien Piedcoq, Gilles Fromentin, Daniel Tomé, Patrick C. Even

**Affiliations:** ^1^UMR914, CRNH-IdF, INRA, Nutrition Physiology and Ingestive Behavior, Paris, France; ^2^UMR914, CRNH-IdF, AgroParisTech, Nutrition Physiology and Ingestive Behavior, Paris, France; ^3^Chaire Aliment, Nutrition, Comportement Alimentaire (ANCA), AgroParisTech, Paris, France

**Keywords:** rat model, obesity prone, glucose, insulin, CCK, dietary obesity, indirect calorimetry, glucose tolerance test

## Abstract

We previously reported that rats prone to obesity exhibit an exaggerated increase in glucose oxidation and an exaggerated decline in lipid oxidation under a low-fat high-carbohydrate (LF/HC) diet. The aim of the present study was to investigate the mechanisms involved in these metabolic dysregulations. After a 1-week adaptation to laboratory conditions, 48 male Wistar rats were fed a LF/HC diet for 3 weeks. During weeks 2 and 3, glucose tolerance tests (GTT), insulin tolerance tests (ITT), and meal tolerance tests (MTT) were performed to evaluate blood glucose, plasma, and insulin. Glucose and lipid oxidation were also assayed during the GTT. At the end of the study, body composition was measured in all the rats, and they were classified as carbohydrate resistant (CR) or carbohydrate sensitive (CS) according to their adiposity. Before sacrifice, 24 of the 48 rats received a calibrated LF/HC meal. Liver, muscle, and intestine tissue samples were taken to measure mRNA expression of key genes involved in glucose, lipid, and protein metabolism. ITT, GTT, and MTT showed that CS rats were neither insulin resistant nor glucose intolerant, but mRNA expression of cholecystokinin (CCK) in the duodenum was higher and that of CPT1, PPARα, and PGC1α in liver were lower than in CR rats. From these results, we make the hypothesis that in CS rats, CCK increased pancreatic secretion, which may favor a quicker absorption of carbohydrates and consequently induces an enhanced inhibition of lipid oxidation in the liver, leading to a progressive accumulation of fat preferentially in visceral deposits. Such a mechanism may explain why CS rats share many characteristics observed in rats fed a high-glycemic index diet.

## Introduction

Large differences are observed between individuals in their capacity to properly adjust substrate oxidation and energy expenditure in order to achieve a stability of body weight and body composition in the long term. This is particularly the case in situations of easy access to palatable energy dense foods (1–5). High-fat (HF) diets, when compared to high-carbohydrate (HC) diets, are more at risk to induce body weight gain because of the large storage capacities of adipose tissue and the low satiating effects of high fat diets, as compared to the low capacities of the glycogen stores and of the *de novo* lipogenesis cost (6). Moreover, HC diets have been proposed as a way to fight against obesity (7–9), and recent dietary guidelines insist on reducing fat intake and maintaining 45–65% of intake as carbohydrate based on whole grain, vegetables, and fruits (10). However, the idea of replacing fat with carbohydrate is now challenged by the respective quality of fats and carbohydrates (11), insisting on the adverse effects of high glycemic index carbohydrate on the control of food intake and on the evolution of insulin resistance (12, 13). In this context, slowing down the rate of glucose absorption by adding insoluble fibers to a high sucrose diet was shown to reduce fat deposition and alleviate the evolution of the symptoms of metabolic syndrome (14).

**Figure 1:**
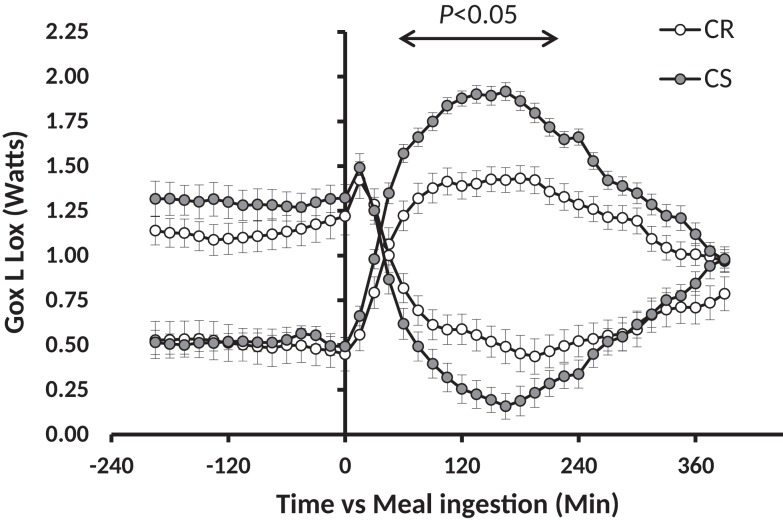
**Meal-induced changes in glucose and lipid oxidation after ingestion of a low-fat high carbohydrate test meal in CR and CS rats**. This figure has been drawn with data taken from the experiment previously published in Nadkarni et al. (17).

Previous studies have shown that feeding rats with a standard low-fat HC starch based diet [conforming to the AIN-93 recommendations for rodents (15)], which was expected to induce a moderate glycemic index, was able to generate differences in adiposity gain, although partly hidden by rather small differences in overall body weight gain (16–18). In these conditions, adiposity does not reach the levels observed with HF diets, but the relative differences of gain prone vs. resistant animals are as large as or even larger than observed with HF diets. In addition, fat tends to accumulate more viscerally than in response to HF feeding which can have negative effects in terms of development of metabolic syndrome. Moreover, rats prone to obesity under low-fat HC diets [carbohydrate sensitive rats (CS)] are not necessarily fat sensitive (FS) (meaning prone to obesity under an HF diet); in fact, only 50% are suggesting that the underlying metabolic defects leading to carbohydrate or fat sensitivity to obesity may be different (17). Accordingly, analysis of the feeding and activity behavior of these animals and in-depth analysis of the various components of energy expenditure revealed that both CS and FS rats exhibit a defective pattern of spontaneous activity when they are fed with the diets to which they are sensitive (16, 18). Moreover, only the CS rats reveal an exaggerated increase in glucose oxidation and an exaggerated decline in lipid oxidation in response to an HC meal (Figure 1) (17). In the longterm, such a response could explain the progressive accumulation of fat in CS rats.

The goal of the present study was to identify the mechanisms responsible for the exaggerated meal-induced changes in glucose and lipid oxidation in CS rats. It was hypothesized that in these CS rats, the insulin response to feeding and/or the sensitivity to insulin is greater than in carbohydrate resistant (CR) ones. To that purpose, Wistar rats were submitted for 3 weeks to HC feeding, during which their response to glucose, meal, and insulin tolerance tests were measured. At the end of the study, muscle, liver, and intestine were sampled for quantification of mRNA expression 2 h after ingestion of a calibrated test-meal.

## Materials and Methods

### Experimental procedure

The study was approved by the French National Animal Care Committee (number 12/098) and conformed to the European legislation on the use of laboratory animals. Forty-eight male Wistar rats were ordered at 7 weeks of age (Harlan) and were delivered in groups of eight at 2-week intervals. They initially weighed 229.8 ± 1.5 g. All the rats were submitted to the same dietary procedure: after a 1-week adaptation to laboratory conditions (temperature 22°C ± 1, humidity 60%, 12/12 L/D cycle lights on at 08:00), during which they were fed a standard laboratory chow (A04, SAFE, France), they were switched to a semi-synthetic LF/HC diet for a 3-week period (UPAE, INRA, Joey-en-Josas, France). It is important to note that this diet was close in macronutrient composition to the standard A04 diet and conformed to the AIN-93 recommendations (Table 1), and therefore that the rats were not submitted to significant changes in the macronutrient composition of their diet during the study. The use of this semi-synthetic diet allowed us to closely control and secure the stability of the origin of the macronutrient sources throughout the study.

**Table 1:** **Macronutrient composition of the diet**.

	HCD
**Weight content (g/kg)**
Milk proteins	140.0
Starch	622.4
Sucrose	100.3
Soy Oil	40.0
Minerals	35.0
Vitamins	10.0
Cellulose	50.0
Choline	2.3
**Energy content (%)**
Protein	14.7
Carbohydrate	75.9
Fat	9.4
Energy density (kJ/g)	15.95
Food quotient	0.946

*The diet was prepared by the “*atelier de préparation des aliments,”* UPAE, INRA, Jouy-en-Josas, France. Energy density is computed assuming a metabolizable energy of 16.7 kJ/g for carbohydrates and proteins, and 37.7 kJ/g for fat. Food quotient is computed assuming a quotient of oxidation of 1.0 for carbohydrates, 0.825 for proteins, and 0.70 for lipids*.

At the end of the 3 weeks of regimen, the rats were killed and their body composition precisely measured by dissection and weighing of the main organs and tissues.

In the first three groups (*n* = 24, initial weight 231.7 ± 2.1 g, final weight 338.1 ± 7.2 g), a glucose tolerance test (GTT) and an insulin tolerance test (ITT) were performed in random order during the second and third week of the regimen (only one test in each week). The GTT was performed in 6 h-fasted rats as indicated for a standard GTT, and ITT was performed on fed rats. In 15 of these 24 rats, respiratory exchanges in response to a GTT were also followed by indirect calorimetry. At least, 1 week of washout was ensured between the two tests in the rats that underwent the two procedures. In the last three groups (*n* = 23, initial weight 228.8 ± 2.1 g, final weight 316.3 ± 4.2 g), a 60 kJ meal tolerance test (MTT) of the regimen was performed during the second or third week of the regimen. The MTT was performed after an overnight fast to ensure that the rats ingested the test meal within 30 min after presentation.

### Glucose and insulin response to GTT, ITT, and MTT

For GTT, food was removed at 08:00 in the morning. Glucose 1.5 g/kg (solution 0.25 g/ml) was injected i.p. at 14:30 and blood samples (200 μl) were taken from the tail vein ~15 min before (t0), then 10, 20, 30, 60, 90, and 120 min after injection. For ITT, insulin 0.3 U/kg dissolved in 1 ml 0.9% NaCl was injected in fed rats at 14:00. Blood samples were taken from the tail vein before (t0), then at 10, 20, 30, 60, and 90 min after injection. For MTT, rats were fasted overnight. A calibrated test-meal (60 kJ) of the diet was given to the rat at 10:00, and it was visually controlled that the rats ingested the meal within 30 min. Blood was taken from the tail vein ~15 min before (t0), then at 60, 120, 180, 240, and 300 min after meal onset. In all studies, glucose was immediately assayed using a standard glucometer (Life-scan, One touch Vita). Blood was centrifuged (10 min, 3000 *g*, 4°C) and plasma stored at −20°C for subsequent assay of insulin. Plasma insulin was detected using enzyme linked immunoassay (Mercodia Rat Insulin, ELISA).

### Changes in the rates of glucose and lipid oxidation in response to GTT

The rats were housed in a calorimetry cage at 18:00 and were fasted overnight while in the calorimetry cage. Water was available throughout. Recording of respiratory exchanges was immediately started. The next day at 10:00, the rats received 1.5 g/kg glucose i.p. and were immediately returned to the metabolic cage. Measurements of respiratory exchanges were continued until 17:00. Glucose oxidation (Gox) and lipid oxidation (Lox) were computed from resting oxygen and carbon dioxide consumption (VO_2_ and VCO_2_) with the following formula (19): Gox (Watts) = [(4.57 * VCO_2_)–(3.23 * VO_2_)] * (15.6/60), Lox (Watts) = [(1.69 * VO_2_)–(1.69 * VCO_2_)] * (39.5/60), with VO_2_ and VCO_2_ in mL/min, 15.6 = kJ/g for glucose, 39.5 = kJ/g for lipids. Division by 60 is to convert Joules/min into Joules/second = Watts. For a detailed description of the calorimetry procedures, see Ref. (19).

### Euthanasia, tissue collection, and segregation between CR and CS rats

Euthanasia was performed at the end of the third week of regimen by i.p. injection of an overdose (60 mg/kg) of sodium pentobarbital. Body composition was assessed by dissection and weighing of the main organs and tissues (liver, spleen, carcass, pancreas, subcutaneous, epididymal, retroperitoneal and mesenteric adipose tissues, skin, heart, scapular brown adipose tissue). In order to perform an objective segregation between CR and CS rats, we used the Student’s confidence interval of the final adiposity values (fat weight/body weight) of the rats computed at the 1% level with Excel. Mean observed adiposity was 9.57% with a standard deviation (SD) of 2.12% and a Student’s confidence interval (SCI) of 0.831%. We thus classified as CR the rats with an adiposity below 8.74% (Mean–SCI) (*n* = 13), and as CS the rats with an adiposity above 10.40% (Mean + SCI) (*n* = 18). The other rats (*n* = 16) were removed from data analysis.

For analysis of mRNA expression in the liver, muscle, and intestine, the nutritional status of the 23 rats of the last three groups was normalized by an overnight food deprivation followed by a 60 kJ meal given 2 h before euthanasia. Euthanasia was performed by decapitation after gas anesthesia with isoflurane (3% in 2 L/min). For analysis of mRNA expression, pieces of liver, gastrocnemius muscle, and intestine were rapidly collected and frozen in liquid nitrogen. Trunk blood and liver samples were also collected for TG content determination. Intestinal mucus of duodenum, jejunum, and ileum were collected as follows: after dissection of the pancreas and mesenteric adipose tissues, the intestine was rapidly removed and separated into three parts (duodenum, jejunum, and ileum). The pieces of intestine were flushed with sterile PBS, and epithelial cells were collected by scraping on ice, placed in TRIzol^®^, and frozen in liquid nitrogen. After thawing, cells were disrupted with a needle.

### Gene expression profile measurement

#### RNA preparation and gene expression measurement

Total RNA was extracted using TRIzol^®^ reagent (Invitrogen, Carlsbad, CA, USA). RNA concentration was assessed using a nanodrop spectrophotometer at 260 nm and RNA integrity was confirmed by electrophoresis on agarose gel. Retrotransciption was performed on 0.4 μg of RNA for muscle and liver or on 10 μg of RNA for intestine to synthetize cDNA using High Capacity cDNA Archive Kit Protocol (Applied Biosystems).

Real Time PCR was performed to measure gene expression on an ABI 7300 (Applied Biosystems) using Power SYBR GREEN PCR MIX (Applied Biosystems). The primer sequences of genes were designed with Primer Express software and the sequence of each primer is given in Table 2. We studied mRNA encoding proteins involved in metabolism such as glycolysis [Glucokinase (GK), Liver-pyruvate kinase (L-PK), Hexokinase 2(HK2)]; lipogenesis [Acetyl-coA carboxylase (ACC), Fatty acid synthase (FAS)]; fatty acid oxidation (Peroxisomal acyl-coenzyme A oxidase 1 (ACOX1), Carnitine palmitoyltransferase 1a-liver isoform (CPT1a), Carnitine palmitoyltransferase 1b-muscle isoform (CPT1b), Fatty acid translocase (CD36), Peroxisome proliferator-activated receptor gamma coactivator 1-alpha (PGC1α), Peroxisome proliferator-activated receptor alpha (PPARα); branched-chain amino acid metabolism [branched chain amino-acid transaminase 2 (BCAT2), 3-methyl-2-oxobutanoate dehydrogenase (BCKDH)]; proteolysis [Ubiquitin-conjugating enzyme E2B (UBE2B), Cathepsin D (CaD)]. For intestine, we studied the expression of the anorexigenic gut peptides cholecystokinin (CCK), peptide YY (PYY), glucagon-like peptide-1 (GLP-1), and glucose-dependent insulinotropic polypeptide (GIP) in the different sections of intestine.

**Table 2:** **Primer sequences used for liver, muscle, adipose tissue, and intestine mRNA analysis**.

Gene	Sequence
GK	forward 5′-TTGAGACCCGTTTCGTGTCA – 3′
	reverse 5′-AGGGTCGAAGCCCCAGAGT -3′
L-PK	forward 5′-TGATGATTGGACGCTGCAA – 3′
	reverse 5′-GAGTTGGTCGAGCCTTAGTGATC – 3′
HK2	forward 5′-AACCGAACAAGCTGGTGTAC-3′
	reverse 5′-TGCACACATCTATAGGTGGC-3′
ACC	forward 5′-CAACGCCTTCACACCACCTT -3′
	reverse 5′-AGCCCATTACTTCATCAAAGATCCT -3′
FAS	forward 5′-TGCTCCCAGCTGCAG -3′
	reverse 5′-GCCCGGTAGCTCTGGGTGTA -3′
ACOX1	forward 5′-AAGAAATCCCCACTGAACAAAACA -3′
	reverse 5′-CCCAGGGAAACTTCAAAGCTT -3′
CPT1a	forward 5′-ATATCAAGGACAGCAGGCACAT -3′
	reverse 5′-CTCAGCAGCCTCCCATGCT -3′
CPT1 b	forward 5′-CAGCCATGCCACCAAGATC -3′
	reverse 5′-CTTGGGCAGTGATGTTTGGA -3′
CD36	forward 5′-CAGCCTCCTTTCCACCTTTTG-3′
	reverse 5′-AAGGCGTTGGCTGGAAGAA-3′
PGC1α	forward 5′-ATACCGCAAAGAGCACGAGAAG-3′
	reverse 5′-CTCAAGAGCAGCGAAAGCGTCACAG-3′
PPARα	forward 5′-GGGATGAAGAGGGCTGAGC-3′
	reverse 5′-TGATTAACATTGGGCCGGTT-3′
BCAT2	forward 5′-GGCGGACCCTTCATTCGT-3′
	reverse 5′-TTCCCCCCCAACTTGCA-3′
BCKDHα	forward 5′-CCAGGGTTGGTGGGATGAG-3′
	reverse 5′-GGCTTCCATGACCTTCTTTCG-3′
UBE2B	forward 5′-AACGCAGTTATATTTGGACCAGAAG-3′
	reverse 5′-ACGGTTGGTGGTTTATTTGGAT-3′
CAD	forward 5′-CGCAGTGTTTCACAGTCGTCTT-3′
	reverse 5′-TGGACTTGTCACTGTTGTACTTATGG-3′
CCK	forward 5′-CAGGTCCGCAAAGCTCCTT3′
	reverse 5′-TCCAGGCTCTGCAGGTTCTT-3′
PYY	forward 5′-CGGCAGCGGTATGGAAAA-3′
	reverse 5′-TGTGAAGAGCAGTTTGGAGAACA-3′
GLP-1	forward 5′-CTCCCGCCGTGCTCAA-3′
	reverse 5′-TTGTTCCGGTTCCTCTTGGT-3′
GIP	forward 5′-CTGCTGGTGCTCCTGTTCCT-3′
	reverse 5′-CATGGGATCGGAACTCAACCT-3′

*The forward and reverse primers were designed using primer express software (Applied Biosystems). The following abbreviations are used: GK, glucokinase; L-PK, liver-pyruvate kinase; HK2, hexokinase 2; ACC, acetyl-coA carboxylase; fatty acid synthase (20); ACOX1, peroxisomal acyl-coenzyme A oxidase 1; CPT1a, carnitine palmitoyltransferase 1a-liver isoform; CPT1b, carnitine palmitoyltransferase 1b-muscle isoform; CD36, fatty acid translocase; PGC1α, peroxisome proliferator-activated receptor gamma coactivator 1-alpha; PPARα, peroxisome proliferator-activated receptor alpha; BCAT2, branched chain amino-acid transaminase 2; BCKDHα, 3-methyl-2-oxobutanoate dehydrogenase; UBE2B, ubiquitin-conjugating enzyme E2B; CaD, cathepsin D; CCK, colecystockinin; PYY, peptide YY; GLP-1, glucagon-like Peptide-1; GIP, glucose-dependent insulinotropic polypeptide*.

RT-PCR was performed using 10 ng (5 μl) of cDNA for liver, muscle, or adipose tissue, and 250 ng (5 μl) of cDNA for intestine in addition to 15 μl of the reagent mix containing RNAase free water, PCR Mix plus forward and reverse primers as previously described (21). The threshold (CT) was set with the constant value for all of the genes and samples to quantify the mRNA concentration, and gene expression was calculated as: 2^−ΔCT^, where ΔCT = CT Gene – CT 18S. Data are means ± SE expressed as a percentage of the values of the CR rats. Negative controls were used to detect potential contamination (control without RT or RNA).

### Statistical analysis

47 of the 48 rats completed the study. In addition, rat 43, which was the fattest one (adiposity 15.87%) with a very high subcutaneous fat deposition and plasma TG values below mean − 2SD, and rat 30, (adiposity 7.99%, would have qualified as CS) with liver and plasma TG values above mean + 2SD, were excluded from the study.

Results are reported as mean ± SEM. Between group comparisons were done by Student’s *t*-tests in Excel. Changes in blood glucose and plasma insulin induced by the GTT, ITT, and MTT were analyzed by ANOVA with Statgraphics^®^ V5.1. Differences with *P*-values below 0.05 were considered as significant.

## Results

### Body weight and body composition

**Figure 2:**
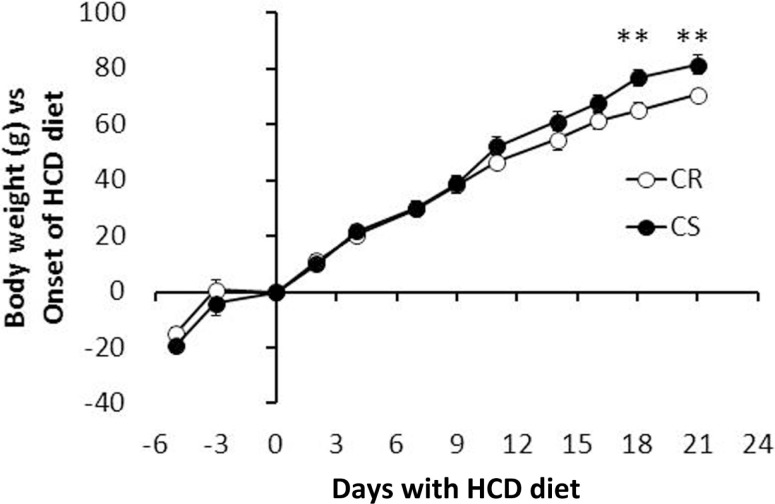
**Evolution of body weight: CR and CS rats had similar body weight at the onset of the study**. Differences developed slowly and did not become significant before 21 days (at the time rats were ~10 weeks old).

The main elements of body composition are given in Table 3. CS and CR rats had similar BW at the onset of the study. At the end, CS rats were significant but only 9.6% heavier than CR rats. Evolution of BW during the study (Figure 2) indeed showed that differences in BW between CS and CR rats became significant only after 21 days of the regimen. Lean body mass (LBM) and carcass mass were not different but CS rats had much larger masses of all the adipose tissues and greater adiposity levels (+62%). The ratio of visceral to subcutaneous fat was not lower in CS than in CR rats, indicating that excess fat mass accumulated in the viscera as much as subcutaneously. Among the organs, only liver weight was significantly increased in CS rats.

**Table 3:** **Body composition of the CR and CS rats (data in g except otherwise stated)**.

	CR (Mean ± SEM)	CS (Mean ± SEM)	*P*
Init weight	230.7 ± 2.0	232.0 ± 2.5	0.726
Final weight	310.4 ± 5.0	340.5 ± 7.9	0.006
Delta weight	79.7 ± 4.1	108.42 ± 6.2	0.001
Carcass	143.2 ± 2.7	146.9 ± 3.4	0.417
Skin	52.5 ± 0.9	57.8 ± 3.3	0.018
Total fat	21.78 ± 0.80	39.92 ± 1.41	<10^−11^
Subcutaneous	9.37 ± 0.63	16.00 ± 0.61	<10^−7^
Mesenteric	2.53 ± 0.16	4.74 ± 0.18	<10^−8^
Epidydimal	4.82 ± 0.24	8.23 ± 0.49	<10^−5^
Retroperitoneal	5.05 ± 0.30	9.85 ± 0.55	<10^−6^
Adiposity (%)^a^	7.03 ± 0.27	11.39 ± 0.15	<10^−14^
Ratio visc/subc^b^	1.39 ± 0.11	1.47 ± 0.10	0.602
LBM	209.3 ± 3.3	220.0 ± 5.0	0.110
Liver	8.54 ± 0.28	10.20 ± 0.39	0.004
Spleen	0.583 ± 0.016	0.618 ± 0.016	0.142
Heart	0.727 ± 0.014	0.769 ± 0.018	0.091
kidneys	2.01 ± 0.05	2.13 ± 0.06	0.178
Testis	3.46 ± 0.12	3.66 ± 0.17	0.377
Pancreas	1.08 ± 0.06	1.05 ± 0.06	0.728
Scapular BAT^c^	0.600 ± 0.040	0.683 ± 0.027	0.083
Adrenals	0.062 ± 0.003	0.060 ± 0.004	0.659

^a^Adiposity = total fat mass/body weight * 100.

*^b^(Epipidymal + retroperitoneal + mesenteric fat)/subcutaneous fat*.

*^c^BAT, brown adipose tissue*.

### Blood glucose and plasma insulin responses to glucose, insulin, and meal tolerance tests

GTT and ITT were performed on the 24 rats of the three first groups, among which six were *a posteriori* classified as CR (final adiposity 7.22 ± 0.28%) and 10 as CS (final adiposity 11.60 ± 0.21%). MTT were performed on the 24 rats of the last three groups, among which eight were *a posteriori* classified as CR (final adiposity 7.00 ± 0.41%) and eight as CS (final adiposity 11.82 ± 0.60%).

**Figure 3:**
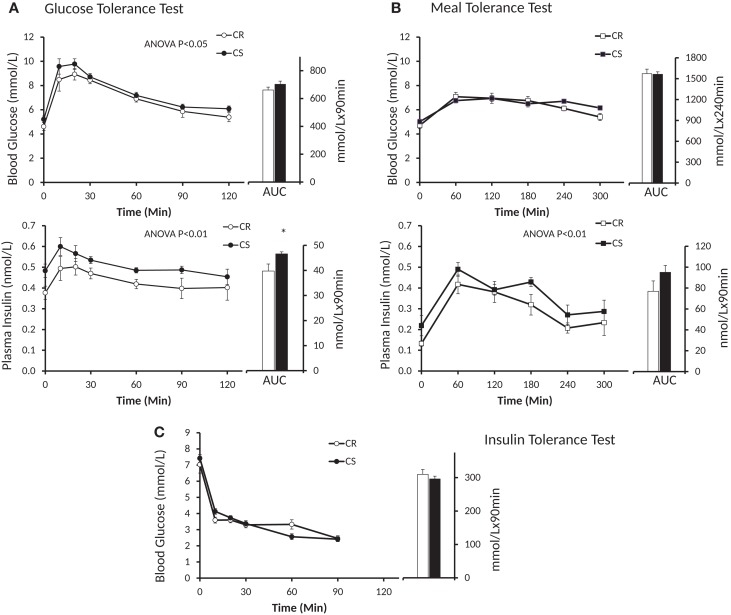
**Changes in blood glucose and plasma insulin induced by the GTT (A), MTT (B) and ITT (C)**. Open symbols, CR rats; black symbols, CS rats; AUC, area under curve. Blood glucose during GTT: *P* = 0.02, Group * Time = 0.99; blood glucose during MTT: *P* = 0.27, Group * Time = 0.25; plasma insulin during GTT: *P* = 0.0016, Group * Time = 0.81; plasma insulin during MTT: *P* = 0.009, Group * Time = 0.90.

Insulin levels and HOMA indices were higher in CS rats (Table 4) before the GTT, i.e., after 6 h fast, but not before the MTT, i.e., after an overnight fast. Figure 3 displays the glucose and insulin responses to GTT, ITT, and MTT. The decline in plasma glucose after ITT (Figure 3C) did not reveal differences between CR and CS rats, indicating that CS rats were not resistant to the hypoglycemic effect of insulin. Glucose and insulin levels during GTT were significantly higher in CS than in CR rats (Figure 3A). During MTT, only insulin levels were significantly higher (Figure 3B). Areas under the curve were significantly different only for the insulin response to GTT. When basal blood glucose or plasma insulin values were introduced as a covariate in the ANOVA, no more differences were observed between groups, indicating that differences during GTT and MTT were due to preexisting differences in basal glucose and insulin levels.

**Table 4:** **Basal blood glucose (mmol/L), plasma insulin (nmol/L), and HOMA index {[glucose (mmol/L) × insulin (pmol/L)]/22.5} at the onset of the glucose and meal tolerance tests**.

	CR (Mean ± SEM)	CS (Mean ± SEM)	*P*
**Onset of GTT**			
Glucose	4.62 ± 0.32	5.21 ± 0.27	0.223
Insulin	0.366 ± 0.024	0.483 ± 0.032	0.013
HOMA	37.9 ± 4.18	57.5 ± 5.90	0.041
**Onset of MTT**			
Glucose	4.70 ± 0.26	5.01 ± 0.21	0.372
Insulin	0.132 ± 0.013	0.219 ± 0.049	0.132
HOMA	14.4 ± 2.08	23.7 ± 4.36	0.088

#### Changes in Glucose and Lipid Oxidation in Response to GTT

**Figure 4:**
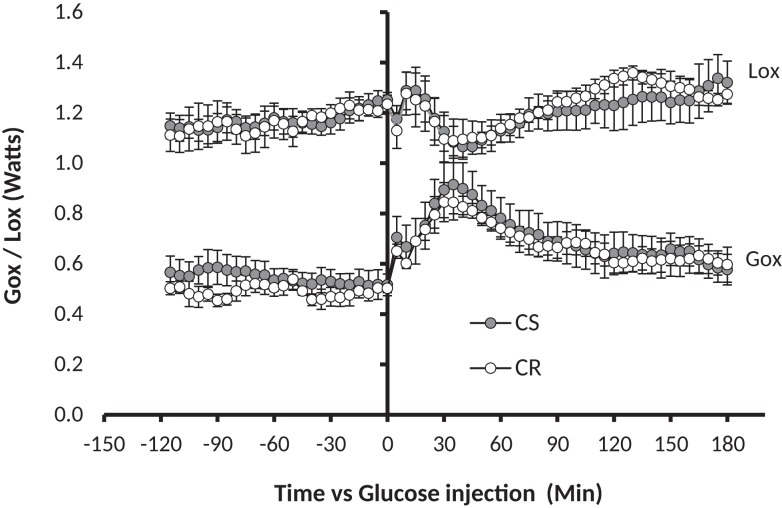
**GTT-induced changes in glucose oxidation (Gox) and lipid oxidation (Lox)**. Glucose 1.5 g/kg (solution 250 mg/ml) was injected i.p. at t0 (see Materials and Methods). Note in comparison to Figure 1, the 10-fold difference on the *y*-axis, the twofold difference on the time axis, and the fact that the MTT affected both Gox and Lox while the GTT affected Gox much more than Lox.

Changes in glucose and lipid oxidation in response to GTT were measured by indirect calorimetry on 15 rats, among which four were *a posteriori* classified as CR (final adiposity 6.53 ± 0.44%) and six as CS (final adiposity 11.26 ± 0.2%). The GTT induced an increase in glucose oxidation that peaked between 30 and 60 min, then returned to basal levels within 120 min (Figure 4), a kinetic that closely paralleled the changes observed in blood glucose (Figure 3). Changes in Lox mirrored the changes in Gox but were of smaller amplitude and duration.

#### Postprandial Expression Profile of Genes Involved in Metabolism in Liver, Muscle, and Intestine 2 h After Ingestion of a Test-Meal

##### Genes involved in glucose oxidation and de novo lipogenesis

**Figure 5:**
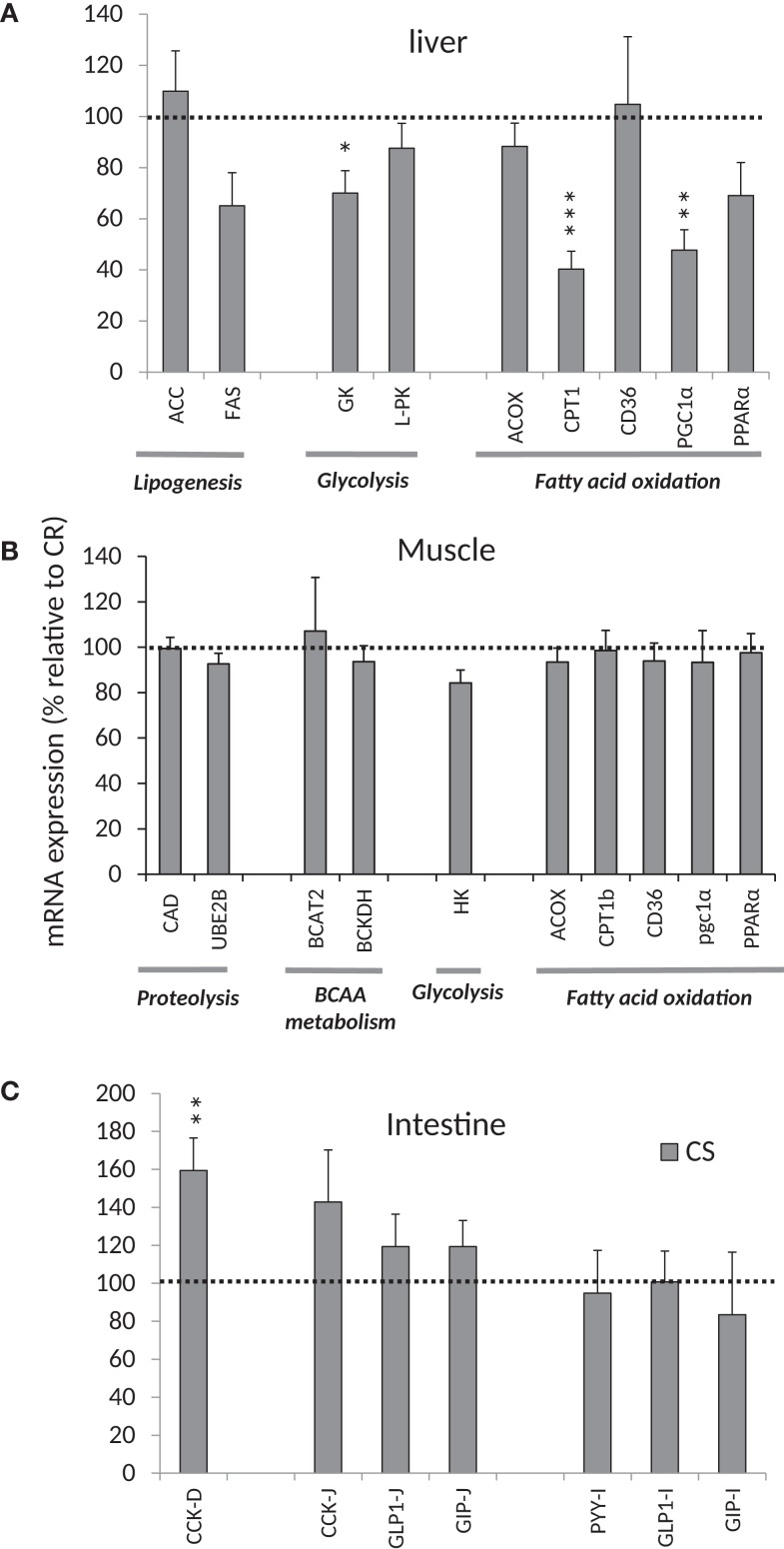
**mRNA expression relative to mRNA expression in CR rats**. **(A)** mRNA expression for enzymes/transporters involved in lipogenesis, glycolysis, and FFA oxidation in the liver, **(B)** mRNA expression for enzymes/transporters involved in proteolysis, BCAA metabolism, glycolysis, and FFA oxidation in muscles, **(C)** mRNA Expression of peptides involved in satiety signaling in the intestine. *P < 0.05, **P < 0.01, ***P < 0.001. See Table 2 for abbreviations.

In the liver (Figure 5A), GK gene expression was 40% lower in CS rats, suggesting that glycolysis may be reduced, but gene expression of L-PK situated downstream of GK was not different. On the other hand, expression of genes involved in lipogenesis such as ACC FAS did not differ between CS and CR rats. Taken together, these results suggest that the control of glycolysis and lipogenesis in liver did not differ significantly between CR and CS rats.

In muscle, no significant changes were observed for HK2, the first enzyme of glycolysis (Figure 5B).

##### Genes involved in lipid oxidation

In the liver (Figure 5A), expression of PGC1α (a transcriptional coactivator regulating genes involved in energy metabolism and in particular mitochondrial biogenetics) was twice as high in CR rats. Expression of PPARα (a transcription factor and key regulator of genes involved in multiple processes of lipid metabolism) also tended to be higher in CR rats than in CS rats (*P* < 0.08). In turn, mRNA encoding CPT1a (which is under the control of PPARα and PGC1α (22) and controls the transfer of long-chain fatty acyl CoA into mitochondria) was 2.5-fold greater in CR rats. Other genes involved in fatty acid oxidation in liver, such as ACOX1 and CD36, were unchanged. These results suggest that CS rats exhibit a lower potential for fatty acid oxidation in liver.

In muscle (Figure 5B), no difference was observed for the expression of genes involved in the control of fatty acid oxidation as indicated by lack of differences in the expression of PGC1α and CPT1b.

##### Branched-chain amino acid metabolism and proteolysis in muscle

We observed no difference in the expression of genes involved in proteolysis (CAD and UBE2B) and BCAA metabolism (BCAT2 and BCKDH) in muscle (Figure 5B).

##### Gene expression in the duodenum, jejunum, and ileum

mRNA expression of CCK was larger in duodenum and jejunum of CS rats but significant only in duodenum (Figure 5C). No differences were observed in the mRNA expression of GLP1, PYY, and GIP.

#### TG in Blood and Liver

Plasma TG measured 2 h after ingestion of a 60 kJ test meal was significantly higher in CS rats [mmol/L: 2.46 ± 0.13 (*n* = 7) vs. 2.015 ± 0.13 (*n* = 7), *P* = 0.03) as was liver TG (mg/g: 13.23 ± 0.52 (*n* = 7) vs. 11.19 ± 0.39 (*n* = 7), *P* < 0.01]. There was also a significant correlation between liver TG and adiposity (*R*^2^ = 0.59, *n* = 14, *P* < 0.01), plasma TG and adiposity (*R*^2^ = 0.457, *n* = 14, *P* < 0.01), and plasma TG and liver TG (*R*^2^ = 0.561, *n* = 14, *P* < 0.01).

## Discussion

The main goal of this study was to investigate the mechanisms contributing to the exaggerated increase in glucose oxidation together with an exaggerated decrease in lipid oxidation observed after ingestion of an HC test-meal in CS rats (17). These changes were suspected to support the progressive increase of fat mass in CS rats. Our initial hypothesis was that CS rats could be more sensitive to insulin and/or produce more insulin in response to ingestion of a high carbohydrate meal.

In this study, Wistar rats were fed a standard high-carbohydrate low-fat diet designed according to the AIN93 recommendations (15), in which most of the carbohydrate was provided as starch, and was thus expected to have a moderate glycemic index. Measurements were performed very early, between 2 and 3 weeks after the onset of feeding with the experimental diet, because the goal was to search for mechanisms that may be responsible for, and not derive from, the experimentally induced changes in body weight and body composition.

Analysis of body weight and body composition confirmed previous reports that CS rats can hardly be discriminated from their body weight gain (16, 17); at the end of the study, CS rats weighed only 9% more than the CR ones. Furthermore, LBM was not significantly different between the two groups. In contrast, total fat was 82% greater and rather evenly distributed between the subcutaneous (+76.5%), mesenteric (+83%), epididymal (+67%), and retroperitoneal (+93%) depots. From unpublished MRI measurements collected in similar conditions, we extracted a group of 12 rats weighing the same weight (232.1 ± 1.8 g), as the rats of this study at the onset of the experiment, and observed that in these rats, fat mass amounted from 9.6 to 18.7 g (13.8 ± 0.87 g). From this data, it can be estimated that during the 3 weeks of the study, CS rats accumulated about three times more fat than CR ones. Among the organs, only liver weight increased significantly but the increased TG content was far too small to explain this difference.

The phenotype of the CS rat is different from that of FS rats [in which the increase in body weight gain and fat mass is much higher, both fat and LBM masses are increased, subcutaneous adipose tissue expands more than visceral, and there is no difference in liver TG content after 3 weeks of HF feeding (18)]. All these differences suggest that the mechanisms responsible for the accumulation of fat are different in CS and FS rats. We also previously reported that contrary to FS rats, CS rats are not hyperphagic but display increased rates of glucose oxidation and decreased rates of in lipid oxidation after ingestion of a high carbohydrate meal (17). In CS rats, the early increase in liver TG, the correlation of plasma and liver TG with adiposity levels, and the high proportion of fat stored viscerally suggest that the primary metabolic defect may come from an increased conversion of lipids into TG in the liver. Since visceral fat is more diabetogenic than subcutaneous fat, this may induce the development of insulin resistance and metabolic syndrome at lower adiposity levels than with HF diets (23, 24). The clinical implications of these observations are important, since obesity is often associated with consumption of diets too high in fat, and strategies most often orient patients toward the consumption of a diet higher in carbohydrate. Considering the possibility that some individuals may react negatively to diets high in carbohydrate because of an accelerated absorption of glucose, such a systematic strategy should be reconsidered in particular in patients with visceral obesity. The results of this study need to be confirmed and completed; the mechanisms possibly responsible for an accelerated rate of glucose absorption suggested by the results of the present study should be investigated, in particular the essential functions of the endocrine and exocrine pancreas in which, for example, mutant leaky type2 ryanodine receptors lead to impaired glucose homeostasis and decreased fuel-stimulated insulin release (25).

The similarity of the decrease in blood glucose after insulin injection during the ITT clearly indicates that CS rats are as sensitive as CR ones to the glucose-lowering effect of insulin, and thus are not resistant to insulin. On the other hand, we observed that plasma insulin and the HOMA index were higher in the CS rats before the GTT after the rats were fasted 6 h, but the differences were much reduced and no more significant before the MTT after the rats were fasted overnight. Taken together with the results of the ITT, these insulin levels and HOMA indices suggest that plasma insulin, and to a lesser extent, blood glucose levels, decrease more slowly in CS than in CR rats but that CS rats still did not suffer significant insulin resistance at the time of the study. Differences were also observed in glucose and insulin values after the GTT and MTT. However, these differences were primarily due to preexisting differences in the basal state before the tests: if, rather than absolute levels, one considers the amplitudes of the glucose and insulin response, they were similar in CR and CS rats. In addition, using indirect calorimetry, we did not observe significant differences in the changes in glucose and lipid oxidation induced by the GTT. This lack of difference sharply contrasts with the large and very significant differences in Gox and Lox previously reported in response to ingestion of a meal-test (17). The reason for this difference possibly has its roots in the amount and rate of delivery of energy induced by the GTT and the MTT, and therefore the respective role of the liver and peripheral tissues in the handling of the caloric load: the early and large increase in peripheral blood glucose after the GTT indicated that much glucose escaped metabolism by the liver and was probably oxidized peripherally by muscles. Accordingly, indirect calorimetry showed that over 3 h, the GTT increased Gox but did not significantly affect Lox (Figure 4), suggestive of an uncoupling between the respective rates of Gox and Lox as expected if glucose oxidation had occurred in the liver. By contrast, after the MTT, we observed that peripheral blood glucose increased much less and we previously reported that the decrease in lipid oxidation quite mirrored the increase in glucose oxidation (Figure 1), indicating that the handling of the glucose derived from the meals occurred primarily in the liver (17). The similarity of changes in glucose and lipid oxidation in CR and CS rats after the GTT, where glucose was directly injected in the abdominal cavity, and the differences after ingestion of the meals, where most of the glucose was produced by the degradation of ingested starch, suggest that the differences in post-meal rates of glucose and lipid oxidation between CR and CS rats depend on initial differences in the degradation of starch in the intestine and/or in differences in the speed of absorption of glucose by the intestine.

mRNA expression of genes coding for enzymes involved in glucose and lipid metabolism in liver may depict a possible mechanism to explain the increased Gox and decreased Lox in CS rats after a MTT. We indeed observed that in liver, 2 h after ingestion of the calibrated test-meal, mRNA expression of several enzymes involved in fatty acid oxidation, such CPT1, PPARα, and PGC1α, was lower in CS rats, suggesting that at this time lipid oxidation may be lower in CS rats as observed *in vivo* by indirect calorimetry (17). By contrast, no differences were observed in mRNA encoding genes involved in lipogenesis, i.e., FAS and ACC, but this lack of response may also be due to the fact that measurements were performed exactly 2 h after ingestion of the test-meal, so at a time when glycogen stores were still not fully restored. Indeed, hepatic lipogenesis is under the control of a complex system and can be stimulated only when there is substrate availability and glycogen stores have been replenished (26). However, liver and plasma TG measured 2 h after ingestion of the MTT were greater in CS rats and generally correlated with adiposity, which suggest that lipid synthesis was higher in the liver of CS rats. Assuming that the CS rats were not insulin-resistant, their small but regularly higher insulin levels may have reduced the expression of liver mRNA encoding proteins involved in fatty acid oxidation, thus reduced lipid oxidation and favored TG release in the blood and accumulation in the liver. In muscle, the enzymes involved in the control of protein, glucose, and lipid metabolism were not affected, which agrees with the fact that we observed no differences in Gox and Lox after the GTT that induced large changes in peripheral blood glucose. This also agrees with the fact that we previously observed no differences in the meal-induced changes in the respiratory quotient of working muscles (17). Taken together, these results suggest that in CS rats, the primary mechanism responsible for the increased adiposity may be an insulin-induced exaggerated decrease in the rate of lipid oxidation by the liver, which makes more free fatty acids (FFA) available for TG synthesis and storage in the liver and visceral adipose tissues.

The higher insulin levels in CS rats in the basal state as well as in response to the GTT and MTT could be related, at least for a part, to the significantly higher expression of intestinal CCK mRNA that may reflect increased CCK release. Protein and fat (27–29) and to a lesser extent carbohydrates (30, 31) stimulate CCK release. Each of these hormones enhances insulin secretion and improves glucose tolerance. Both CCK-8 and CCK-32 are potent stimuli for insulin release as measured *in vitro* and in animal models (32, 33). In addition, CCK stimulates secretion of digestive enzymes by the pancreas, among which amylase, which may favor an accelerated degradation of starch and absorption of glucose by the intestine. Therefore, even with starch-based diets, such rats may evolve as if maintained on a higher glycemic index (GI) diet compared to CR rats. At the rather young age when they were studied (10 weeks old), the rats still responded normally to insulin but fasting insulin levels and HOMA indices already tended to be increased. Thus in the long-term, it is possible that CS rats could develop significant insulin resistance and glucose intolerance as observed in rats fed a high GI diet. By contrast, it was previously reported that impaired lipid oxidation in response to high GI diets occurs before development of impaired insulin secretion and insulin resistance (34), which was indeed the case in the rats of this study and was also suggested by the more pronounced decrease in whole body lipid oxidation reported previously (17). Rats and mice fed low-fat high GI diets are not hyperphagic, do not gain more weight, or only marginally more, but accumulate more fat and predominantly more visceral fat (35–37). They develop insulin resistance without impaired glucose tolerance within 8–12 weeks (13, 35), and in the longer term, 8–52 weeks, exhibit higher basal insulin levels and impaired glucose tolerance (35, 36, 38, 39). Results on blood TG are contradictory; higher (38) on a high GI low-fat diet or lower (13) on a 30% fat Western type diet, but liver TG is consistently increased. All these characteristics fit with what we observed in the CS rats in the present and previous studies, and may be responsible for the cascade of events finally leading to the sensitivity to obesity of CS rats fed a standard high-carbohydrate diet.

## Conclusion

The CS rat model shows that obesity is not necessarily dependent upon high-fat feeding and/or overfeeding. This study points to the possible involvement of an exaggerated CCK response to ingestion of an HC diet possibly responsible for an accelerated degradation of starch in the intestine and increased delivery of glucose into the blood leading CS rats to reduce fat oxidation in the liver and progressively develop insulin resistance and visceral obesity in a process similar to the one observed in rats fed a high GI diet. The consistency of the CS rat model and the observation that such a form of obesity also seems to occur in the mouse model (40) suggest that this phenotype may also be observed in humans. If the hypotheses raised here are confirmed, one should consider extending this research into humans. As recently described to diagnose lactose intolerance in the rat model than in humans (41, 42), measurements of changes in glucose and lipid oxidation in response to ingestion of a high-carbohydrate meal should be considered as a non-invasive test to assess in humans the predisposition to gain excess fat with a low-fat high-carbohydrate regimen.

## Conflict of Interest Statement

The authors declare that the research was conducted in the absence of any commercial or financial relationships that could be construed as a potential conflict of interest.

## Abbreviations

ACC, acetyl-coA carboxylase; ACOX1, peroxisomal acyl-coenzyme A oxidase 1; BCAT2, branched chain amino-acid transaminase 2; BCKDHα, 3-methyl-2-oxobutanoate dehydrogenase; CaD, cathepsin D; CCK, colecystockinin; CPT1a, carnitine palmitoyltransferase 1a-liver isoform; CPT1b, carnitine palmitoyltransferase 1b-muscle isoform; CD36, fatty acid translocase; CR, carbohydrate resistant; CS, carbohydrate sensitive; FAS, fatty acid synthase; FR, fat resistant; FS, fat sensitive; GIP, glucose-dependent insulinotropic polypeptide; GK, glucokinase; GLP-1, glucagon-like peptide-1; GTT, glucose tolerance test; HC, high carbohydrate; HF, high fat; HK2, hexokinase 2; ITT, insulin tolerance test; LF, low-fat; L-PK, liver-pyruvate kinase; MTT, meal tolerance test; PGC1α, peroxisome proliferator-activated receptor gamma coactivator 1-alpha; PPARα, peroxisome proliferator-activated receptor alpha; PYY, peptideYY; UBE2B, ubiquitin-conjugating enzyme E2B.
